# Letter from Dr. Beaugrand

**Published:** 1839-08-10

**Authors:** 


### FOREIGN CORRESPONDENCE.

### LETTER FROM DR. BEAUGRAND.

PARIS, *June 9th*, 1839.

The medical community of Paris has just witnessed one of the most remarkable scientific discussions which has for a long time taken place, before the Royal Academy of Medicine.

The advantages of the experimental method, *exclusively* employed in physiology, the doctrine of Charles Bell, relative to the division of nerves into sensitive and motor, have just been questioned and powerfully shaken by M. Gerdy, notwithstanding M. Blandin's able defence of them. Now that the discussion has terminated, let us give a retrospective glance, and observe what results we may arrive at.

We stated that there were two litigated points. 1. The precise definition of the sense to be accorded to the phrase, *experimental method*, and the physiological value to be attached to it. 2. The specifications of the functions of the nervous system. Let us examine, successively, these two questions.

What is to be understood by the term *experimental method?* Physiologists have, as yet, come to no agreement on this subject. Some, with MM. Bouillaud and Rochaux, wish to express by it the sum total of the means necessary to arriving at the discovery of the truth; that is to say, observation, ratiocination, direct experiments, &c. Others, M. Gerdy at their head, contend that it is the art of interrogating nature, that she may be compelled to answer; in a word, the art of experimenting in physics and in chemistry, and of physiological vivisections. Observation, then, with them, consists simply in the application of the senses to the study of phenomena which are spontaneously produced: whilst experience is in the sum of knowledge acquired by divers means. We willingly assent to this latter opinion, and, in fact, the experimental method is and can be nothing more than a method based on experiment, and all agree in considering this as synonymous with vivisection. Let us cease, then, to confound things so essentially different, and let us see what experiments can teach us in physiology.

When a living animal is dissected, it is necessary, in order to examine an organ whose functions re to be studied, to cut or remove the tissues concealing this organ from the eye of the physiologist. He may readily believe, then, that the severity of the injury inflicted must exercise an unfavourable influence over the wished for results. How do we know that the integrity of the mutilated parts is not requisite for the accomplishment of the normal phenomena?

This applies especially to experiments upon the cerebral and spinal nerves; besides, can we compare that which goes on in the animal, amid such tortures, with what is taking place in man during health or sickness? It is, to say the least, doubtful, when we reflect that the subjects of these experiments are usually selected from the inferior orders of animals. The organization of some, like the batracians, differs greatly from ours. Have we not repeatedly witnessed frogs and other reptiles surviving the most frightful mutilations? Have we not seen geese walking and swimming for some moments after sudden decapitation? and if we descend to the bottom of the zoological scale, among the polypi, actineans and star-fish, do we not find these animals enjoying sensation, not only without the intervention of a brain, but even without any appreciable nervous system. Thus, comparative anatomy and physiology can throw but a feeble light upon human physiology. Here, as M. Gerdy remarks, it becomes a vacillating and treacherous guide.

But if any thing can demonstrate the uncertainty attending vivisections, it is, without doubt, the diversity of results obtained by experiments upon one and the same question. This last remark enables us, without farther delay, to enter upon the important discussion concerning the nervous system; a subject which has been agitated for such a length of time, and which is still involved in obscurity.

First, however, let us rapidly review the opinions of ancient authors upon this matter. The first idea of distinguishing the nerves into sensitive and motor, belongs to the Alexandrian school. The testimony of Galen is precise on this particular, (De. Loc. Affect. lib. iii. cap. ult.) It had been noticed that in paralysis, sensation and motion might be lost separately or together, and this phenomenon had aroused the attention of observers; they asked themselves by virtue of what anatomical conditions this could take place; but the subject baffled all the talent of Herophilus and Endemius. Galen, in that portion of his work devoted to physiology, (De Usu Partium, lib. viii.) establishes, in opposition to the opinion of Aristotle, that the brain is the source of voluntary movement and of sensation, and that two orders of special nerves preside over these two faculties. The nerves of sensation and and of the senses are softer, and spring from the anterior portions of the encephalon, (cerebrum,) while the nerves of motion are harder and less tortuous, and arise at the posterior and most consistent part, (the cerebellum.) It would appear from some obscure phrases, interrupted by numerous digressions, that he believed the nerves of sensation penetrated deeply into the central soft part, whilst the motor nerves emerged directly from the firmer and denser surface. These ideas were reproduced by the Arabs, (Aricenna, lib. i. p. 59,) with the exception of Averrhoes, who maintained the ancient doctrine of Aristotle, and continued to regard the heart as the centre of the faculties, which Galen, with the Alexandrian school, referred to the brain. The doctrine of the physician of Pergamus, found in the sixteenth century an energetic supporter in the anatomist, Desmoulins. The great Fernel has slightly modified the Galenic theory on this point, as on every other. He thinks that the nerves of the senses spring from the anterior part of the cerebrum, and the motor nerves from the posterior, but he adds that those which preside over the sense of touch originate in the membranes. In favour of this notion he alleges that the brain, being insensible, cannot perceive the sensation of touch, while the membranes, being endowed with a lively sensitiveness, have an aptitude for receiving these impressions, (De Anima Facult. lib. v. cap. x.)

The emptiness of these distinctions was afterwards discovered, and Van Swieten, in his Commentaries on Boerhaave, (b. iii. p. 343 and 455, ed. in 4to.) after having proved that sensation and motion must have nerves of different origins, admits that they are entirely unknown.

I will not detail the ideas of the moderns on the distinction of the nervous system of animal functions, and of that of the functions of relation, as it has no connection with the subject under consideration; so that I finally arrive at Charles Bell. This excellent physiologist says, that the nerves may be ranged into three classes; those of the first class, arising from the anterior columns of the spinal marrow, presiding over motion; the second, coming from the posterior columns, transmitting sensation; whilst the third, given off by the lateral tracts, reserves to itself the direction of special movements—those of respiration. The anatomical data on which Charles Bell founded his system, as well as the experiments which seemed to sustain it, are well known; we will not, therefore, repeat that with which every one is acquainted. Magendie, in France, arrived at analogous results by the too exclusive method of verisection, and was induced to admit as true, first, the distinction between the motor nerves (anterior) and the sensitive, (posterior,) but not so entirely as the English physiologist had advanced; secondly, that the theory of the respiratory nerves was false: this constitutes the first and remarkable difference in their opinions.

On the other hand, Bellingheri employed himself in the same researches, and following the experimental plan, was led to proclaim that there existed an antagonism of motive action in the anterior and posterior columns of the spinal marrow; the first, presiding over extension, the second over flexion. That sensation resided in the cortical substance, and motion in the medullary. Schapp, Herbert, Mayo, M.M. Fodera, Calmeil, Gerdy, and others, have obtained still more discordant results. Such was the state of the question when M. Blandin roused the debate of which we have to give some account, and which has occupied the Academy during nearly two months.

Three orders of facts, anatomical data, experiments and physiology have been invoked, each in its turn, with a view to attack or defend the speciality of action of the nervous system. These we will review in order.

It is said that there is a perfect similitude between the spinal nerves and the fifth pair.

All have two roots; one with a ganglion, *for sensation;* the other without, *for motion;* thus the spinal nerves and the facial regulate these two orders of phenomena.

Let us examine this first proposition; but, before doing so, we should notice that the filamentous origins of the spinal nerves do not spring from the central portion of the spinal marrow, but from the most superficial layer, in such a way, that, according to some anatomists, Desmoulins among others, the apparent union is merely a juxtaposition.

Is the case the same as regards the fifth pair? Assuredly not. Several of the most respectable authors, Gall, Meckel, Cloquet, &c., assign three roots to it; but let us admit that it has actually but two, the one superficial, the other deep seated. The radicles which compose the first, or motive branch, do not come from the anterior portion of the spinal marrow, but, from the *tuber annulare;* those radicles, by the junction of which the ganglionary or sensitive root is formed, dive deeply into the *tuber* and divide into two fasciculi, one of which proceeds transversely to reach the aqueduct of Sylvius, while the other plunges vertically into the substance of the corpus restiforme.

These origins are very variable. Now, if, as every one admits, the nerves owe their properties to the parts where they arise, can it be said that the spinal nerves and the fifth pair are similar? for, if we have recourse to the functions for the establishment of this pretended similitude, how happens it, we ask, that a division of the trigeminus produces on the other nerves of the senses such remarkable effects? paralysing, as it does, the smell, taste, and vision; inducing, too, acute ophthalmia, a loss of sight, whilst a section of the spinal nerves produces merely paralysis of the sensation and touch. It can be shown, moreover, that the olfactory and optic nerves do not come from the posterior part of the spinal marrow; and also, that notwithstanding their simplicity of action, they have a complex origin; finally, that the different movements of the eye have relation not only with the anterior portions of the spinal marrow, but with other and very different parts of the encephalon.

M. Blandin has observed, that, in man, the nerves of the thoracic members have the posterior root more developed, relatively to the anterior, than the nerves of the pelvis, and hence concludes that this disposition has reference to the fact, that the sensibility of the superior members is greater than that of the inferior. We might content ourselves with saying that M. Blandin has reasoned in a vicious circle, having taken for granted that which in truth has to be proved; but it is better to examine the questions themselves.

In the first place, is it a fact that the members are organs of sensation rather than of motion? secondly, do the inferior members possess less sensitiveness than the superior? Authors have greatly exaggerated the sensitiveness of those parts destined for touch; the human hand, for example, being rather an *organ of motion.* This doctrine appears paradoxical, because it is at variance with certain ideas which have been received without due examination; but let us reflect a little, and ask ourselves whether we do not use the hand more as an instrument of prehension than of tact. Is not the hand, in all professions, more frequently employed in seizing, grasping, and turning, than in feeling? and if the hand is better adapted for this last action than any other portion of the body, is it not owing to its form, to the number and proximity of its articulations one with another, which allow of its intimate application to all bodies, and thus enables it to appreciate every variety of form and density?

This conducts us directly to the second question. It is quite erroneous to suppose that the superior members are more sensitive than the inferior. The pulp of the fingers is not in the least affected by that tickling which is intolerable to the soles of the feet or the sides. Is the sensitiveness of the hand comparable to that of the genital organs or anus? yet the ganglionary roots which are distributed to these last mentioned parts are smaller than those of the thoracic members. The anatomical remark, then, of M. Blandin, far from befriending the partisans of Charles Bell, is, on the contrary, completely hostile to them.

But are the experiments more favourable to them? The rapid exposition which we have made of the results which the labours of Charles Bell, MM. Magendie, Bellingheri, Schapp, Herbert, Mayo, and the contradictory ones of MM. Fodera, Calmeil, and others, have led to, have already answered in the negative, it being impossible to reconcile with each other the facts reported by them. We have elsewhere seen what amount of importance is to be attached to vivisection and comparative anatomy.

Much stress has been laid on the following experiment of Charles Bell, as already demonstrating the distinction, into motor, and sensitive nerves. On irritating the posterior roots of the spinal nerves in an animal recently killed, no result was obtained, whilst the same treatment directed to the anterior roots produced motion. Charles Bell performed this experiment but once. But supposing the effect to be constant, what does it prove? The animal *is dead*; there is no longer *any communication between the brain and spinal marrow.* Should we hence conclude that the posterior roots are devoted exclusively to sensation and the anterior to motion? Surely not, and Charles Bell has taken care not to make so sweeping a deduction. He closes his narration of this experiment by saying, that the anterior and posterior columns of the spinal marrow have *different functions.* He, then, does not look upon the question as settled by this single fact. (Charles Bell, Expos. du Systéme, Nat. Paris, 1825. p. 17.)

Let us turn now to the pathological facts. Not to speak of the numerous and well authenticated cases of *complete* destruction of the spinal marrow within a limited space, in which the portions of the body *below* the injury *retained* sensation and motion, several instances are reported of considerable softening of the anterior columns, unfollowed by paralysis of motion, and of similar lesion of the posterior column where sensation was unaffected. The work of M. Ollivier (d'Angers) on diseases of the spinal marrow, contains several very curious examples of this kind. What conclusion, then, must we draw? That Charles Bell's system is entirely false? Certainly not. It possesses some truths of the deepest interest, but which must be taken in a less absolute sense than they have been heretofore. These are precisely the truths which have gained this doctrine all its credit. Thus, for instance, the facial is a motor nerve, rather than a nerve of sensation, yet it is not entirely destitute of sensitiveness, for it has repeatedly exhibited symptoms of this where it emerges from the stylo mastoid foramen, and the sensibility of the anostamotic filaments which it receives from the fifth pair, below the irritated spot, cannot be adduced in explanation of this; we may add, too, that several cases of facial neuralgia are on record which cannot be accounted for by the slender communication afforded by the trigeminus. As to the functions of the spinal marrow, nothing has been actually demonstrated; perhaps, at some future period, the functions of the different component parts of this organ may be discovered; so far, however, opinions and facts are equally at variance.

We hear it said, and incessantly repeated, that the system of Charles Bell can alone explain partial paralysis of sensation or motion.

From the most remote antiquity, this phenomenon has excited the attention of medical men, and each one has presented us with his theory. The one which appears to me the least objectionable is that of Mercurialis. One or more of the different faculties of organ, he says, may undergo change independently of the other; now, sensation and the property of communicating motion are two distinct faculties, and can, therefore, be separately destroyed, (Med. Pract. lib. i. cap. xx. p. 113.) All authors, moreover, have remarked, that in the majority of cases motion was the function lost, or was at least soonest affected; it being a very complex phenomenon, and one demanding, more than any other, the integrity of a greater number of parts.

In fine, we have witnessed the theory of Charles Bell at fault, as regards certain facts in which the anatomical lesion did not correspond to the functional disorder.

As the discussion of which we are speaking was about terminating, M. Magendie communicated to the Academy of Sciences the results of some experiments on the nervous system, which I will briefly state.

The spinal nerves of sensation and of motion *are equally sensitive* when both are uninjured.

If the nerve of sensation be cut, the motor nerve instantly loses its sensitiveness. Should the motor nerve be divided in the middle, the extremity which remains attached to the spinal marrow is entirely incapable of sensation; the opposite end, however, preserves it.

In this case the sensibility proceeds *from the circumference to the centre.*

If the nerves of sensation are divided in the middle, the end which remains in contact with the spinal marrow is extremely sensitive, and on the contrary, that position nearest the ganglion loses this faculty entirely.

M. Magendie proposes investigating whether this influence of the sensitive on the motor nerve will not be kept up in the spinal marrow, between its component fasciculi, which may themselves be distinguished into sensitive and motor. All then, again, is reduced to the question, what becomes of the previous experiments which were confidently advanced as sustaining the assertion that the posterior roots are insensible? Perhaps, it may be said they were badly conducted; yet, what shall warrant us in giving the superiority to those now in progress? What judgment are we to form in the midst of such a chaos? We must wait, and hesitate in giving credence to opinions in a department of physiology, of which the foundation is scarcely laid.

